# Clinical application of self-made ENBD drainage tube in the treatment of pancreatic pseudocyst: a case report

**DOI:** 10.3389/fmed.2026.1838825

**Published:** 2026-05-15

**Authors:** Hong Wei, Da Yong Sun, Xiang Yu Wang, Dan Wu

**Affiliations:** Department of Gastroenterology, The Second People’s Hospital of Shenzhen City (The First Affiliated Hospital of Shenzhen University), Shenzhen, China

**Keywords:** case report, endoscopic treatment, pancreatic pseudocyst, self-made ENBD drainage tube, transgastric pancreatic cyst gastrostomy

## Abstract

**Objective:**

To explore the safety and efficacy of the modified endoscopic nasobiliary drainage (ENBD) tube in the treatment of pancreatic pseudocyst.

**Methods:**

A case of pancreatic pseudocyst following acute pancreatitis was reported, which was treated with endoscopic transgastric pancreatic cyst drainage using a modified ENBD tube. A double pigtail-like fixation device was fabricated by technically modifying a standard ENBD tube with standardized fabrication, sterilization and quality control procedures to achieve effective drainage of the cyst. The detailed fabrication and operation steps were recorded to ensure the reproducibility of the technology.

**Results:**

The operation was successful. Postoperatively, the cyst was significantly reduced in size, and the clinical symptoms disappeared. Re-examination by computed tomography (CT) 1 month after surgery showed complete resolution of the cyst, with the patient recovering well and no complications observed. During the 6-month follow-up, the patient had no recurrence of the cyst, good dietary and living conditions, and returned to normal agricultural production.

**Conclusion:**

This study is the first to report the clinical application of the modified ENBD drainage tube for transgastric drainage of pancreatic pseudocyst in China, and the success of the operation confirms that this method is safe, effective, and cost-effective. Compared with commercial plastic stents and metal stents, this modified device has obvious economic advantages on the premise of ensuring therapeutic effect, making it worthy of clinical promotion and application in primary hospitals and resource-limited areas.

## Introduction

Pancreatic pseudocyst is one of the common complications of acute pancreatitis, with an incidence rate of approximately 10–26% (1, 2). Active intervention is required when the cyst diameter exceeds 6 cm, persists for more than 6 weeks, or is accompanied by relevant symptoms (3, 4). With the continuous development and improvement of endoscopic technology, endoscopic cyst drainage has become the preferred treatment for pancreatic pseudocyst due to its advantages of minimal invasiveness, rapid recovery, and fewer complications (5, 6).

Currently, the commonly used endoscopic treatment methods in clinical practice include endoscopic transgastric or transduodenal cyst drainage. Although commercial luminal stents such as lumen-apposing metal stents (LAMS) have shown good therapeutic effects in clinical applications, their high cost largely limits their widespread use in primary hospitals and regions with relatively underdeveloped economic conditions (7, 8). Traditional commercial plastic double pigtail stents are more economical than LAMS, but their clinical application is still limited by certain procurement costs and supply channels in some underdeveloped areas (7, 9). Therefore, exploring an economical and effective alternative treatment method is of great clinical significance.

Endoscopic nasobiliary drainage (ENBD) is a common endoscopic interventional device, which is mainly used for biliary tract drainage in clinical practice, such as obstructive jaundice, cholangitis and other diseases (10). It has the characteristics of soft material, good biocompatibility, low cost and easy access. Based on the structural characteristics of ENBD tube and the clinical demand for pancreatic pseudocyst drainage, we innovatively modified the ENBD tube to form a double pigtail-like fixation device, which can be applied to transgastric drainage of pancreatic pseudocyst and make up for the deficiency of commercial stents in clinical application in resource-limited areas.

This paper reports a case of successful treatment of pancreatic pseudocyst using a self-made ENBD drainage tube, which is the first such report in China. While ensuring good therapeutic effects, this method significantly reduces the treatment cost, and its therapeutic effect is comparable to that of commercial plastic stents, providing an economical and practical treatment option for clinical practice.

## Clinical data

### Patient information

A 45-year-old male farmer was admitted to the hospital with “epigastric pain for more than 1 month, aggravated for 3 days.” One month prior to admission, the patient had a history of obvious binge eating and drinking. After the meal, he developed severe persistent epigastric pain, described as knife-like, radiating to the lumbodorsal region, accompanied by nausea and vomiting. The vomitus contained gastric contents and bile. The patient was previously treated in a local hospital and diagnosed with “acute pancreatitis.” After conservative treatment such as fasting, rehydration, and acid suppression, the abdominal pain was relieved. However, the abdominal pain worsened again in the past 3 days, accompanied by abdominal distension and loss of appetite, so he was transferred to our hospital for further treatment.

The patient had no previous history of chronic diseases such as hypertension or diabetes, and no history of drug allergies. He had a long-term drinking history of about 20 years, with an average daily consumption of 200–300 mL of Baijiu (Chinese spirits). The patient’s economic condition was poor, and he could not afford the cost of commercial LAMS and imported plastic stents after communicating with his family.

### Physical examination

The patient was in general good condition, conscious, with no jaundice of the skin or sclera. Abdominal examination: the abdomen was slightly distended, with obvious tenderness in the epigastric region, no rebound tenderness, Murphy’s sign negative, liver and spleen not palpable under the costal margin, and normal bowel sounds. No positive signs were found in cardiopulmonary and neurological examinations.

### Laboratory examinations

Blood routine examination showed: white blood cell count 12.3 × 10^9^/L, neutrophil ratio 78.5%, hemoglobin 135 g/L, platelet count 258 × 10^9^/L. Biochemical examination showed: serum amylase 245 U/L (normal value <100 U/L), lipase 458 U/L (normal value <60 U/L), total bilirubin 18.5 μmol/L, direct bilirubin 6.2 μmol/L, alanine aminotransferase 52 U/L, aspartate aminotransferase 48 U/L, blood glucose 6.8 mmol/L, serum creatinine 86 μmol/L, blood urea nitrogen 5.2 mmol/L. Tumor markers (CA19-9, CEA, CA242) were within the normal range, and coagulation function examination was normal.

### Imaging examinations

Abdominal plain and enhanced CT scans (preoperative) showed swelling of the body and tail of the pancreas, blurred peripancreatic fat space, and a cystic lesion with a diameter of approximately 8.5 cm in the body and tail of the pancreas. The cyst wall was smooth and intact, and the contents showed water-like density with a CT value of about 15 HU. No enhancement of the cyst wall was observed on enhanced scanning (Figure 1①). The cyst was closely adjacent to the posterior wall of the stomach. Gastroscopy revealed a localized bulge on the posterior wall of the stomach, with a diameter of approximately 6 cm, smooth surface mucosa, and soft texture, which was presumably caused by cyst compression (Figures 2①, ②). No obvious abnormal changes were found in the gastric and duodenal mucosa except for the localized bulge.

**Figure 1:**
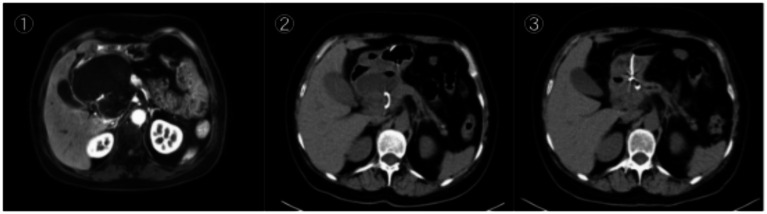
**(①)** Preoperative abdominal enhanced CT suggested a cystic lesion with a diameter of about 8.5 cm in the body and tail of the pancreas; **(②)** abdominal CT performed 2 weeks postoperatively showed a significant reduction in the pancreatic cyst (diameter 3.2 cm); **(③)** abdominal CT performed 4 weeks postoperatively showed a further reduction in the pancreatic cyst (diameter 1.5 cm) with almost complete disappearance of cyst fluid.

**Figure 2:**
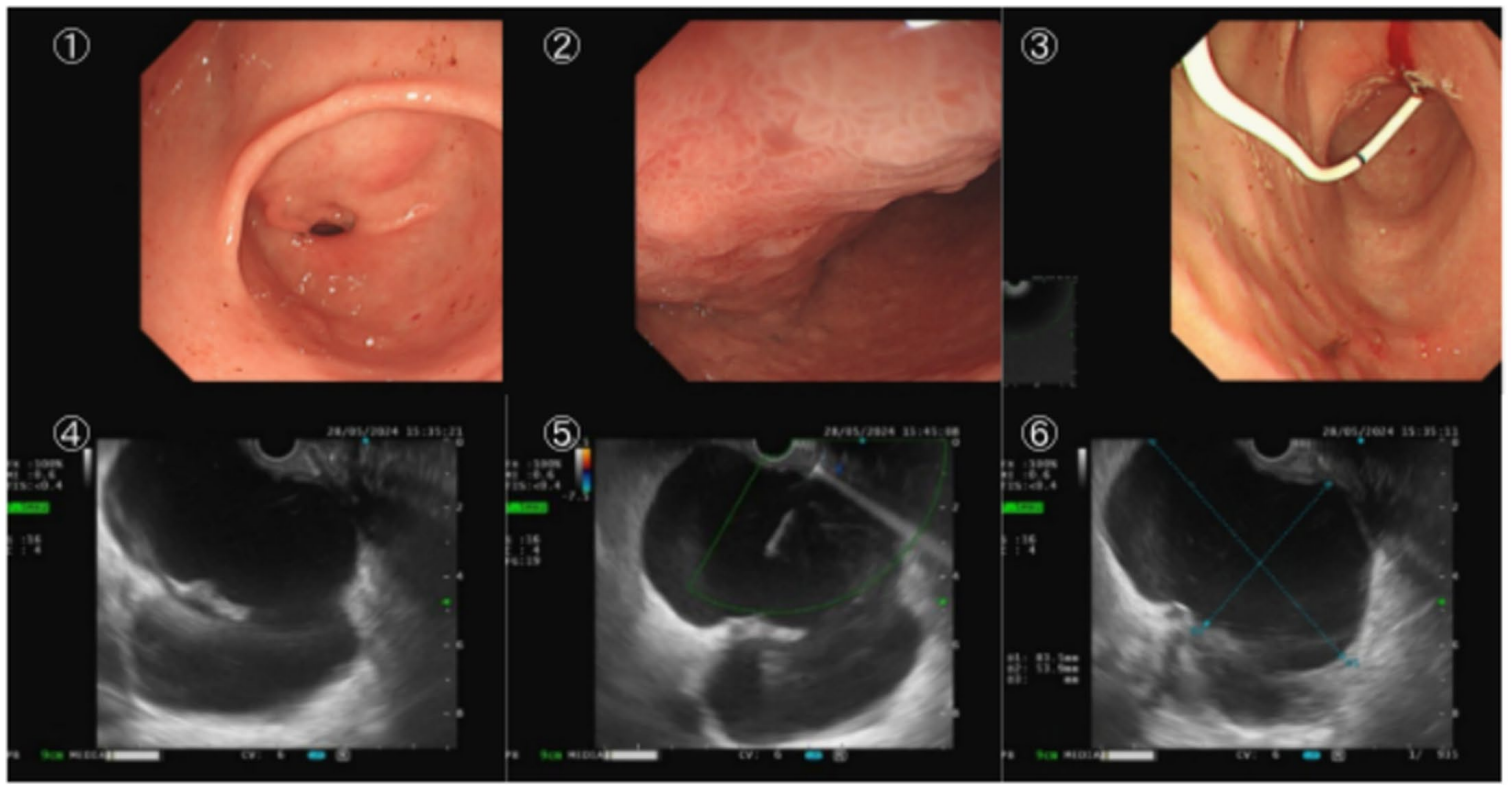
**(①, ②)** Gastroscopy showing the localized bulge on the posterior wall of the stomach caused by pancreatic pseudocyst compression; **(③)** EUS showing the good position of the self-made drainage tube after implantation; **(④–⑥)** endoscopic view showing the puncture, guidewire placement, and drainage tube implantation process of transgastric pancreatic pseudocyst drainage.

### Clinical diagnosis

Pancreatic pseudocyst following acute pancreatitis.

### Treatment method

Considering the large size of the patient’s cyst, obvious symptoms, and close proximity to the gastric wall, endoscopic transgastric cyst drainage was decided. Due to financial constraints, the patient could not afford the expensive cost of commercial metal stents and Plastic Stent, so the modified ENBD drainage tube was used for treatment. The treatment plan was fully communicated with the patient and his family, and written informed consent was obtained.

### Fabrication of the modified ENBD drainage tube

A standard ENBD tube (Olympus curved-tip ENBD tube, specification 7Fr, length 200 cm, material: medical silica gel) was selected as the base material. All fabrication tools were sterilized by high-pressure steam, and the whole fabrication process was completed in a clean bench of the endoscopy center to ensure aseptic operation. The fabrication steps were as follows: first, a 5 cm segment was cut from the proximal part of the distal *α*-loop of the ENBD tube using medical scissors. Secondly, the two ends of the 5 cm segment were bent into a semicircular shape with a diameter of about 1 cm using sterile hemostatic forceps, and the bent parts were fixed with sterile medical nylon thread to form a double pigtail-like structure, which can prevent the drainage tube from migrating into the cyst cavity or falling off from the gastric wall; thirdly, the middle part of the segment was smoothed with sterile sandpaper to avoid mucosal injury during intubation. After fabrication, quality inspection of the self-made drainage tube was performed, including airtightness testing (immersing the tube in sterile normal saline and injecting air to check for air bubbles), flexibility evaluation (bending the tube 180° repeatedly for 10 times to check for cracks) and structural stability testing (pulling the double pigtail part with 5 N force to check for looseness). The qualified drainage tube was sterilized by ethylene oxide and stored in a sealed sterile package for standby.

### Surgical procedure

The patient completed relevant preoperative examinations to rule out surgical contraindications and fasted and abstained from drinking for 12 h before surgery. The operation was performed under general anesthesia, with the patient in the left lateral position. An endoscopic ultrasound (EUS) was inserted through the mouth, first entering the duodenum, then retracting to the gastric cavity to carefully observe the posterior wall of the stomach. An obvious bulge was found on the posterior wall of the stomach (Figures 2①, ②), which was confirmed to be caused by cyst compression combined with preoperative CT images. Endoscopic ultrasound was used to further confirm the relationship between the cyst and the gastric wall, measuring the gastric wall thickness to be approximately 2 mm, and the cyst contents showed anechoic changes (Figure 1①). No obvious blood vessel signals were found in the puncture area by EUS color Doppler, which was suitable for puncture.

Under direct endoscopic vision, the most prominent part of the bulge on the posterior wall of the stomach was selected as the puncture site. A 19G EUS-FNA needle was inserted vertically at this site, and the puncture needle entering the cyst cavity was visualized under ultrasound. Approximately 20 mL of pale yellow clear cyst fluid was aspirated for laboratory testing to confirm the correct puncture position. No contrast agent was injected during the operation, and no cyst cavity washing was performed to avoid the risk of infection and cyst fluid diffusion. The whole operation was completed under the combined guidance of EUS and direct endoscopic vision without X-ray fluoroscopy. A Boston Scientific 0.025-inch yellow zebra guidewire was inserted along the puncture needle, and the guidewire coiling in the cyst cavity was observed under ultrasound. The puncture needle was removed, and a Boston Scientific duodenal papillotome was inserted through guidewire exchange. Under guidewire guidance, a lateral mucosal incision was performed at the puncture site (power 40–100 W, effect 5) until the papillotome penetrated the cyst wall and entered the cyst cavity. The papillotome was withdrawn, and the self-made plastic drainage stent for pancreatic pseudocyst was inserted under guidewire guidance. The stent was released smoothly with a good position, and a large amount of cyst fluid was observed flowing into the gastric cavity (Figures 2④–⑥).

Due to the self-shaping characteristic of the ENBD tube and the artificial double pigtail design, the intragastric end was similar to a pigtail shape, preventing the drainage tube from falling into the cyst cavity. Under endoscopic ultrasound observation, the drainage tube was confirmed to be in a good position (Figure 2③), and the cyst fluid drainage was unobstructed. The operation was performed smoothly without complications such as bleeding. The operation time was about 45 min, and the patient was transferred to the general ward for observation after the operation.

### Treatment results

Immediately after the operation, approximately 200 mL of pale yellow clear cyst fluid was drained through the drainage tube. Biochemical examination of the cyst fluid showed a significant increase in amylase content (820 U/L), which was a direct measurement value of the cyst fluid without dilution. The relatively low amylase level may be related to the long course of the pseudocyst (more than 1 month) and the gradual hydrolysis of amylase in the cyst fluid (11), confirming it as pancreatogenic cyst fluid. On the first day after surgery, the patient’s abdominal pain was significantly relieved. The patient had no fever, abdominal distension or other discomfort, and the vital signs were stable (blood pressure 120/75 mmHg, heart rate 80 beats/min). On the third day after surgery, the patient’s abdominal pain basically disappeared, and he could take liquid food without discomfort such as abdominal pain or fever. The patient’s diet was gradually transitioned to semi-liquid food on the 5th postoperative day, and no adverse reactions were observed. Re-examination by abdominal CT 2 weeks after surgery showed a significant reduction in the cyst size, with the diameter decreasing from 8.5 to 3.2 cm and the cyst wall thickness thinning (Figure 1②).

Re-examination by abdominal CT 4 weeks after surgery showed a further reduction in the cyst diameter to 1.5 cm, with almost complete disappearance of the cyst fluid (Figure 1③). The drainage tube was removed endoscopically on the 30th day after surgery, and the removal process was smooth. Endoscopy during tube removal showed that the gastric wall puncture site had healed well, with no obvious erosion or bleeding, and the pancreatic pseudocyst had basically disappeared. The patient’s clinical symptoms were completely relieved. The patient was discharged from the hospital on the 32nd postoperative day, and the total hospital stay was 38 days.

### Follow-up

The patient was followed up regularly in the outpatient clinic, with follow-up time points of 1, 3, and 6 months after surgery. The patient was followed up in the outpatient clinic 3 months after surgery, with no discomfort such as abdominal pain or distension, normal weight recovery (recovered to the level before onset), and no signs of cyst recurrence on abdominal CT examination. The patient’s serum amylase and lipase returned to normal levels (amylase 85 U/L, lipase 42 U/L). A telephone follow-up 6 months after surgery showed that the patient had a good quality of life, resumed his daily agricultural work, had a regular diet, and quit drinking completely. Abdominal CT re-examination at 6 months showed no cyst recurrence, and the pancreatic morphology was basically normal.

During the entire follow-up period, the patient had no adverse events such as gastrointestinal bleeding, infection, or pancreatic fistula, and the treatment tolerance was excellent. The patient expressed high satisfaction with the treatment effect and low treatment cost, and said that he would actively recommend this method to other patients with similar conditions.

## Discussion

### Treatment strategy for pancreatic pseudocyst

The treatment methods for pancreatic pseudocyst mainly include conservative treatment, percutaneous aspiration and drainage, surgical drainage, and endoscopic drainage (12, 13). For cysts smaller than 6 cm and without symptoms, conservative observation and treatment are usually adopted. However, for cysts with a diameter greater than 6 cm, persisting for more than 6 weeks, or accompanied by complications such as compression symptoms, infection, or rupture, active intervention is required (3, 14). Although traditional surgical treatment has reliable effects, it is associated with large trauma, slow recovery, and many complications. Percutaneous aspiration and drainage have minimal trauma but are prone to infection, have a relatively high recurrence rate (16.3%), and have limited drainage effects when the cyst is not communicating with the gastrointestinal tract (9, 15).

In recent years, with the continuous development of endoscopic technology, endoscopic cyst drainage has become the preferred treatment for pancreatic pseudocyst (16, 17). This method establishes an artificial channel between the cyst and the stomach or duodenum through endoscopy to achieve continuous drainage of cyst fluid, with advantages such as minimal invasiveness, rapid recovery, short hospital stay, and fewer complications. Multiple studies have shown that the technical success rate of endoscopic cyst drainage can reach more than 95%, and the clinical cure rate can reach more than 90% (18).

### Technical advantages of the self-made ENBD drainage tube

The self-made ENBD drainage tube method adopted in this case has multiple technical advantages. Firstly, from a cost-effectiveness perspective, a detailed cost breakdown analysis was conducted: the material cost of the self-made drainage tube is approximately 700 yuan (including ENBD tube 500 yuan, sterile nylon thread 20 yuan, fabrication and sterilization costs 180 yuan); while the price of a commercial covered metal stent exceeds 30,000 yuan, the price of a domestic commercial plastic double pigtail stent is about 3,000–5,000 yuan (7, 9, 19). Compared with LAMS, the cost is reduced by about 99%, and compared with domestic commercial plastic stents, the cost is reduced by about 80–90%, showing a significant cost difference. This remarkable cost advantage makes the method particularly suitable for promotion and application in primary hospitals with relatively insufficient medical resources and economically underdeveloped regions.

Secondly, in terms of technical feasibility, the fabrication process of the modified ENBD drainage tube is relatively simple, requiring no special equipment or complex processing technology, and can be fabricated in ordinary digestive endoscopy centers. Medical staff can master the fabrication technology after simple training (about 2–3 training sessions), indicating good promotion prospects. In addition, ENBD tubes are commonly used devices in endoscopy centers, with stable supply channels and easy access, which can solve the problem of insufficient supply of commercial stents in some areas.

Thirdly, in terms of clinical effects, the patient in this case achieved satisfactory clinical results after treatment with the modified drainage tube. The cyst completely disappeared within 4 weeks after surgery, the patient’s symptoms were completely relieved, and there was no recurrence during 6 months of follow-up. The treatment effect is comparable to that of commercial stents reported in the literature. The modified ENBD tube has soft material and good biocompatibility, which can reduce the damage to the gastric wall and cyst wall during indwelling, and the double pigtail design can effectively prevent stent migration, which is an important guarantee for the success of treatment.

### Technical key points and precautions

The successful treatment of pancreatic pseudocyst with the self-made ENBD drainage tube depends on the following technical key points: Firstly, the selection of indications is crucial. This method is suitable for patients where the cyst is closely adjacent to the gastric or duodenal wall (the distance between the cyst wall and the gastrointestinal wall is less than 5 mm), the cyst wall thickness is less than 10 mm, and the cyst fluid is non-infectious. Caution should be exercised when the cyst is far from the gastrointestinal tract or separated by important blood vessels. Patients with severe coagulation dysfunction, active gastrointestinal bleeding and severe infection are excluded from this method.

Secondly, the selection of the puncture site needs to be precise. Puncture should be performed at the site where the cyst is closest to the gastric wall, avoiding blood vessels. The combined application of preoperative CT and intraoperative endoscopic ultrasound helps to accurately determine the optimal puncture site. EUS color Doppler should be used to detect the puncture area to avoid puncturing blood vessels and causing bleeding complications.

Thirdly, the fabrication of the drainage tube fixation device is the technical key. The pigtail-like device must be able to expand stably in the cyst cavity to prevent the drainage tube from falling off, while not being too hard to avoid damaging the cyst wall. Repeated adjustments are required during fabrication to ensure the appropriate elasticity and expanded shape of the fixation device. The diameter of the double pigtail should be controlled at 1–1.5 cm, which is suitable for most pancreatic pseudocyst cavities and can avoid excessive expansion leading to cyst wall injury.

Fourthly, after the operation, the patient should be observed for abdominal pain, fever, gastrointestinal bleeding, etc., and further endoscopic treatment should be performed if necessary. Postoperative fasting and fluid replacement should be performed for 24 h, and the drainage of cyst fluid should be closely observed. Antibiotics are routinely used for 3 days to prevent infection, and proton pump inhibitors are used to protect the gastric mucosa and promote the healing of the puncture site.

### Potential risks, regulatory and safety considerations

Although the modified ENBD drainage tube has achieved good clinical effects in this case, there are still potential risks in clinical application that need to be paid attention to. Firstly, the modified device is a self-made medical device, and its clinical application needs to comply with the relevant regulations of medical device management in China. The fabrication process must be standardized, and strict aseptic operation and quality control must be performed to ensure the safety of the device. Secondly, during the indwelling period of the drainage tube, there may be risks such as stent migration, mucosal erosion, and mild bleeding, which require close postoperative observation and timely handling. Thirdly, if the cyst fluid is infected, the self-made drainage tube may have insufficient drainage flow, which may lead to the deterioration of infection, so strict indication selection is required.

In terms of regulatory compliance, this modified ENBD drainage tube is only used for individual clinical treatment under the premise of obtaining the informed consent of the patient and the approval of the hospital ethics committee, and has not been used for commercial promotion. If it is to be popularized and applied in a large range, it is necessary to go through the relevant registration and approval procedures of medical devices in accordance with the law to ensure the standardization and legality of clinical application (20). In addition, the hospital has formulated the “Standard Operating Procedure for the Fabrication and Clinical Application of Modified ENBD Drainage Tube” to standardize the fabrication, operation and postoperative management of the device and reduce the occurrence of adverse events.

### Limitations and improvement directions

Despite the many advantages of the modified ENBD drainage tube method, it also has certain limitations. Firstly, this method has high requirements for the operator’s endoscopic technical level, requiring proficient mastery of endoscopic puncture, dilation, and intubation techniques. Secondly, quality control of the modified equipment is relatively difficult, requiring strict fabrication standards and quality inspection procedures. As a single-center case report, the sample size is small, and the long-term efficacy and safety need to be verified by more clinical studies. Fourthly, the modified drainage tube is only suitable for the drainage of single, unilocular pancreatic pseudocysts, and its application effect for multiple or multilocular pseudocysts needs to be further explored.

Future improvement directions include: establishing standardized fabrication processes and quality control standards, formulating a unified quality inspection manual and fabrication operation video to improve the reproducibility and standardization of the device; conducting multi-center prospective studies to further verify the safety and efficacy of the method; expanding the sample size and including patients from different levels of hospitals to evaluate the clinical application effect of the method in different medical resource conditions; exploring more optimized drainage tube designs, such as increasing the drainage diameter of the tube and adding anti-infection coatings, to improve treatment effects and reduce the risk of infection; and establishing a standardized operation technical training system to promote and popularize this technology. In addition, it is planned to carry out relevant research on the modification of ENBD tubes for the treatment of multilocular pancreatic pseudocysts to expand the indication range of the modified device.

### Literature comparison and clinical significance

Multiple foreign studies have reported that the technical success rate of treating pancreatic pseudocyst with LAMS is 95–100%, the clinical cure rate is 90–97%, and the complication rate is relatively low (21). However, the high price of LAMS ($30,000) limits its widespread application in developing countries, while the cost of domestic plastic stents is only about $400–$700 (7, 9). In recent years, domestic scholars have also actively explored low-cost alternative treatment methods, including the use of plastic stents and modified drainage tubes, which have achieved certain results (19, 22). A study by Ge et al. (19) reported that the clinical cure rate of treating pancreatic pseudocyst with commercial plastic stents was 88.9%, and the recurrence rate was 11.1%, with good clinical effects, but the treatment cost was still about 3,000 yuan per case.

The modified ENBD drainage tube method reported in this case reduces the treatment cost by 99% compared with LAMS and 80–90% compared with domestic commercial plastic stents while ensuring good therapeutic effects, which has important clinical promotion value. Especially for regions with relatively scarce medical resources and patients with limited economic conditions, this method provides a feasible treatment option. This method makes full use of the existing common endoscopic devices in clinical practice, realizes the transformation of medical devices from “special use for special purposes” to “multiple uses for one thing,” and provides a new idea for the exploration of low-cost treatment methods for pancreatic pseudocyst.

## Conclusion

The modified ENBD drainage tube is an innovative, safe, effective, and economical treatment method for pancreatic pseudocyst. This method has a simple fabrication process, low cost, good therapeutic effect, and few complications, making it particularly suitable for promotion and application in primary hospitals and economically underdeveloped regions. Compared with commercial plastic stents and metal stents, it has obvious economic advantages on the premise of ensuring the same therapeutic effect, and its material is easy to obtain, which is in line with the clinical needs of resource-limited areas. However, this method has high requirements for the operator’s technical level, requiring strict quality control and standardized operating procedures. In addition, the clinical application of the modified self-made device needs to comply with relevant medical device management regulations and ethical requirements, and the informed consent of the patient must be obtained. It is recommended to conduct larger-scale clinical studies to further verify its long-term efficacy and safety, providing more sufficient evidence for clinical promotion. At the same time, standardized fabrication and operation specifications should be established, and technical training should be strengthened to ensure the safe and effective application of this method. In the future, we will further optimize the device design and standardize the clinical application process to make this method more suitable for clinical promotion and bring more benefits to patients with pancreatic pseudocyst.

## Data Availability

The original contributions presented in the study are included in the article/supplementary material, further inquiries can be directed to the corresponding author.
